# Production of novel diagnostic radionuclides in small medical cyclotrons

**DOI:** 10.1186/s41181-018-0038-z

**Published:** 2018-02-20

**Authors:** Mateusz Adam Synowiecki, Lars Rutger Perk, J. Frank W. Nijsen

**Affiliations:** 1 Radboudumc, Radboud Translational Medicine B.V, Geert Grooteplein 21 (route 142), 6525EZ Nijmegen, The Netherlands; 2 Radboudumc, Dept. of Radiology and Nuclear Medicine, Geert Grooteplein-Zuid 10, 6525GA Nijmegen, The Netherlands

**Keywords:** Radionuclide production, Cyclotron, Technetium-99 m, Radioiodine, Zirconium-89, Copper-64, Gallium-67, Gallium-68, Yttrium-86, Scandium-44

## Abstract

The global network of cyclotrons has expanded rapidly over the last decade. The bulk of its industrial potential is composed of small medical cyclotrons with a proton energy below 20 MeV for radionuclides production. This review focuses on the recent developments of novel medical radionuclides produced by cyclotrons in the energy range of 3 MeV to 20 MeV. The production of the following medical radionuclides will be described based on available literature sources: Tc-99 m, I-123, I-124, Zr-89, Cu-64, Ga-67, Ga-68, In-111, Y-86 and Sc-44. Remarkable developments in the production process have been observed in only some cases. More research is needed to make novel radionuclide cyclotron production available for the medical industry.

## Background

High-purity radionuclides are a key element in the development of radiopharmaceuticals for applications in nuclear medicine. Radionuclides are used in diagnostic and therapeutic radiopharmaceuticals. Often, they can be imaged by Single Photon Emission Computed Tomography (SPECT) or Positron Emission Tomography (PET). Over the last few decades, research groups in both the private and public sector conducted numerous development studies of new radiotracers. This resulted in a constantly evolving radiopharmaceutical market trying to satisfy the needs of the medical society. Many failed introductions of new radiopharmaceuticals resulted from the inability to stand out from the products that are currently available. This is due to little added value or a lack of clinical relevance. Those that are successful in manifesting their benefits then face another barrier. *Zimmermann* et al. described that the road of successful new radiopharmaceuticals is paved with failures because of the many industrial and regulatory constraints (Zimmermann, 2013). The clinically used radionuclides should satisfy requirements such as:

- Physical properties suitable for its application (half-life (T_1/2_), decay mode, emission energy);
- Chemical properties suitable for labeling, with high radiochemical yields and a high radionuclidic purity;
- An acceptable trade-off between the dose received by the patient and the desired effect (image quality or treatment);
- A reasonable price range.

On the other hand, from a radiopharmaceutical industry point of view, a radionuclide selected for production must meet certain criteria to be of interest:

- The production efficiency is acceptable in terms of equipment and personnel burden;
- The starting materials are not too expensive; the supply is secured and remains stable in the long term;
- It offers a high molar activity;
- It is expected to be carrier-free or no-carrier added in order to limit the toxicity;
- Radionuclide production and the post-processing chemistry can be simplified, with a preference for automation (because of *Good Manufacturing Practice* (GMP), radiation safety and costs concerns);
- Its half-life is suitable for logistics;
- There is sufficient demand on the market.

It often happens that the above criteria from both sides are not met, which causes the radionuclide to never reach widespread application. In addition to that, other implications (such as political or economical changes) may discourage the production of certain radionuclides. This is typically the case when government allocated investments or decisions influence the distribution of sources of the radionuclide, e.g. by phasing out a nuclear reactor (Krijger et al., 2013). Currently most hospital radio-pharmacies derive their radionuclides from three types of sources: nuclear research reactors, radionuclide generators and cyclotron facilities (Table 1). It is worth mentioning that the generators still need a reactor or cyclotron source to produce the parent radionuclide. There are also other, much less common ways of producing radionuclides by using linear accelerators (Mang'era et al., 2015), Van De Graaf accelerators (Jones, Robinson Jr., and McIntyre, 1984) or lasers (Bychenkov, Brantov, and Mourou, 2014).

**Table 1:** Common types of radionuclide sources

	Nuclear Reactors	Generators	Cyclotrons
*Principle of production*	Target material inserted in the neutron flux field undergoes fission or neutron activation transmuting into radionuclide of interest	Long-lived parent radionuclide decays to short-lived daughter nuclide of interest. Daughter nuclide elution follows in pre-determined cycles	Target material irradiation by charged particle beams. Inducing nuclear reactions that transmute the material into radionuclide of interest
*Transmutation base*	Neutrons	Decay	p, d, t, ^3^He, α or heavy ion beams
*Advantages*	- Production of neutron rich radionuclides, mostly for therapeutic use - High production efficiency - Centralized production: one research reactor able to supply to large regions or in some cases globally	- Available on site, no need for logistics - Mostly long shelf life - Easy to use - Limited radioactive waste: returned to manufacturer after use	- Production of proton rich elements used as β^+^ emitters for PET scans - Decentralized production allows for back-up chains - High uptime - High specific activity in most cases - Small investment in comparison to nuclear reactor - Little long-lived radioactive waste
*Disadvantages*	- Extremely high investment cost - High operational costs - Considerable amounts of long-lived radioactive waste - Long out-of-service periods - Trouble to back-up in case of unforeseen downtime - Demanding logistics, often involving air transport - Public safety concerns - Non-proliferation treaty concerns	- Supplies in cycles according to possible elution frequency; in-house use must be timed accordingly - Trace contaminants of long-lived parent nuclide in eluted product	- Regional network of cyclotrons and complex logistics needed for short-lived produced radionuclides - Radionuclide production limited depending on installed beam energy

This review article focuses on recent developments in the production of novel diagnostic radionuclides by using small medical cyclotrons. Some of the presented radionuclides can potentially be used in theranostic applications, i.e. a combined diagnostic and therapeutic effect within single application of the radiopharmaceutical.

### Cyclotron produced radionuclides

**Fig. 1:**
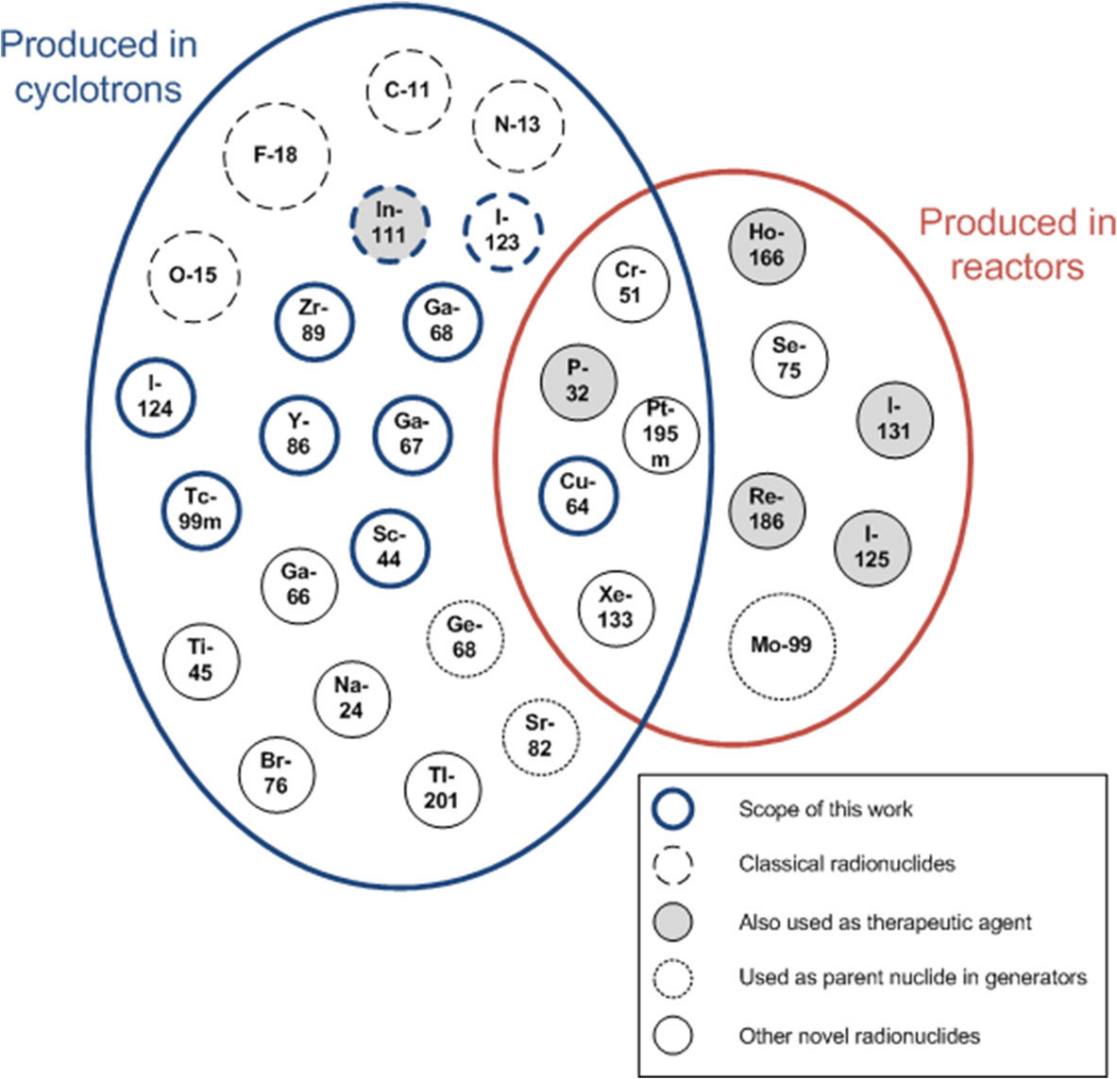
*Radionuclides used in nuclear medicine diagnostics*

*Classical* cyclotron produced radionuclides are defined in this article as produced by very well-established technologies using mostly liquid or gas targets (Fig. 1). Typically, they are produced routinely on an almost daily basis and therefore present the bulk of targetry solutions offered by the cyclotron manufacturers. Among these classical radionuclides are: ^18^F, ^13^N, ^11^C and ^15^O. These four PET radionuclides are also commonly referred to as “*standard*” radionuclides in literature and are mostly produced using low energy medical cyclotrons. ^123^I and ^111^In are two of the cyclotron produced radionuclides used in SPECT studies that can be regarded as classical. However, these are produced by intermediate energy cyclotrons. The *novel* cyclotron produced radionuclides are defined as all others.

Production technology may already be well-established, like ^64^Cu or ^124^I, but what makes them interesting is that they are mostly either produced on an irregular basis or in singular centers across a large area and that their clinical application is not yet established. Most of them are produced using solid target systems with some of them having a proven history of robustness, while others are still under development or require further optimization. Typically, novel radionuclides have relatively longer half-lives which allows for shipment to distant users. The following novel cyclotron produced radionuclides will be discussed (Fig. 1): ^99m^Tc, ^124^I, ^89^Zr, ^64^Cu, ^67^Ga, ^68^Ga, ^86^Y, ^44^Sc. Also, the classical radionuclides of ^123^I and ^111^In are discussed since there are possibilities to use small medical cyclotrons for their production.

### Types of cyclotrons

*Small medical cyclotrons (SMC)* normally have a proton energy below 20 MeV (Table 2). In the literature, they are referred to as *medical cyclotrons*, *PET cyclotrons* or *small-sized cyclotrons*. They offer proton beam currents typically in the range of 60–100 μA. These accelerators are mostly installed in hospitals, universities and small-scale industrial radionuclide production plants. Many of these small medical cyclotrons have been installed over the last two decades and the number is still increasing. In 2008, almost 700 cyclotrons were installed worldwide (IAEA, Cyclotron Produced Radionuclides: Principles and Practice, 2008). Only seven years later, according to *Goethals* et al. (Goethals and Zimmermann, 2015), that number has increased to 1218 cyclotrons whereof approximately 1000 are SMCs (Table 2). Most of the SMCs are located in the developed countries, although newly industrialized developing countries are rapidly increasing their cyclotron base. The majority of these SMCs have been manufactured by four companies in order of market share (Schaffer et al., 2015): General Electric Healthcare (GE Healthcare), Ion Beam Applications (IBA), Siemens and Advanced Cyclotron Systems Incorporated (ACSI). There are several other companies producing small or intermediate energy cyclotrons that are developing steadily (Schmor, 2010). Typically, SMCs accelerate protons only, with some of them also capable of accelerating deuterons at half of the specified energy of protons. Their purpose is the production of medical radionuclides for in-house use, research and commercial purposes. In general, they are focused on the production of short-lived standard radionuclides for PET studies.

**Table 2:** Distinction of cyclotron types (Goethals and Zimmermann, 2015)

Cyclotron type	Energy Range (MeV)	Approximate number	Typical location
Small medical cyclotron (SMC)	< 20 MeV	1050	- hospitals - universities - local commercial plants
Intermediate energy cyclotron	20–35 MeV	100	- regional commercial plants - research institutes
High energy cyclotron	> 35 MeV	50^a^	- research institutes - cancer proton therapy centers

^a^Excluding proton therapy cyclotrons

Cyclotrons delivering protons of an energy between 20 and 35 MeV are considered *intermediate energy* cyclotrons or *medium cyclotrons*. Besides the main proton beam capacity, they usually offer a deuteron beam, and few of them offer an α beam. These machines tend to be located at bigger radiopharmaceutical commercial plants or research institutes. Obviously, they can also be used to produce the classical PET radionuclides, but their main purpose is the production of classical SPECT and novel PET radionuclides, or parent nuclides for generators.

Cyclotrons of particle energies above 35 MeV are considered *high energy cyclotrons.* They are scattered around the globe in well-regarded research institutes. These machines are tailored to specific research needs and can be designed to accelerate many kinds of particles: protons, deuterons, tritium, alpha and heavy ion beams. Many unique novel radionuclides can be produced, especially parent radionuclides for the generators, like ^68^Ge or ^82^Sr. High energy cyclotrons are also installed in large clinical cancer centers for proton beam therapy.

### Cyclotron targetry

The most important irradiation parameters determining the formation of a radioactive product are the beam flux, energy and irradiation time as well as number of target nuclei, nuclear reaction cross section and half-life of the produced radioisotope (Krijger et al., 2013; Qaim, 2017). Standard radionuclides are mostly produced in either *gas* or *liquid* targets. These targets are very easy to use and require no manual handling for routine production activities. The fluidic nature of the irradiated element makes rapid heat exchange possible. Provided water cooling has the potential to remove the heat produced by the intensity of the beam up to the maximum specification of the cyclotron. Often, helium cooling is used for the *beam window,* i.e. the target element through which the beam enters the isolated target material within the target body. Just after End of Bombardment (EOB), the unloading process is performed by pressurized inert gas that pushes the produced radioactivity through small bore tubing to the automatic synthesis module. Therefore, the gas and liquid target methods are safe, reliable and fast.

### Solid targets

**Fig. 2:**
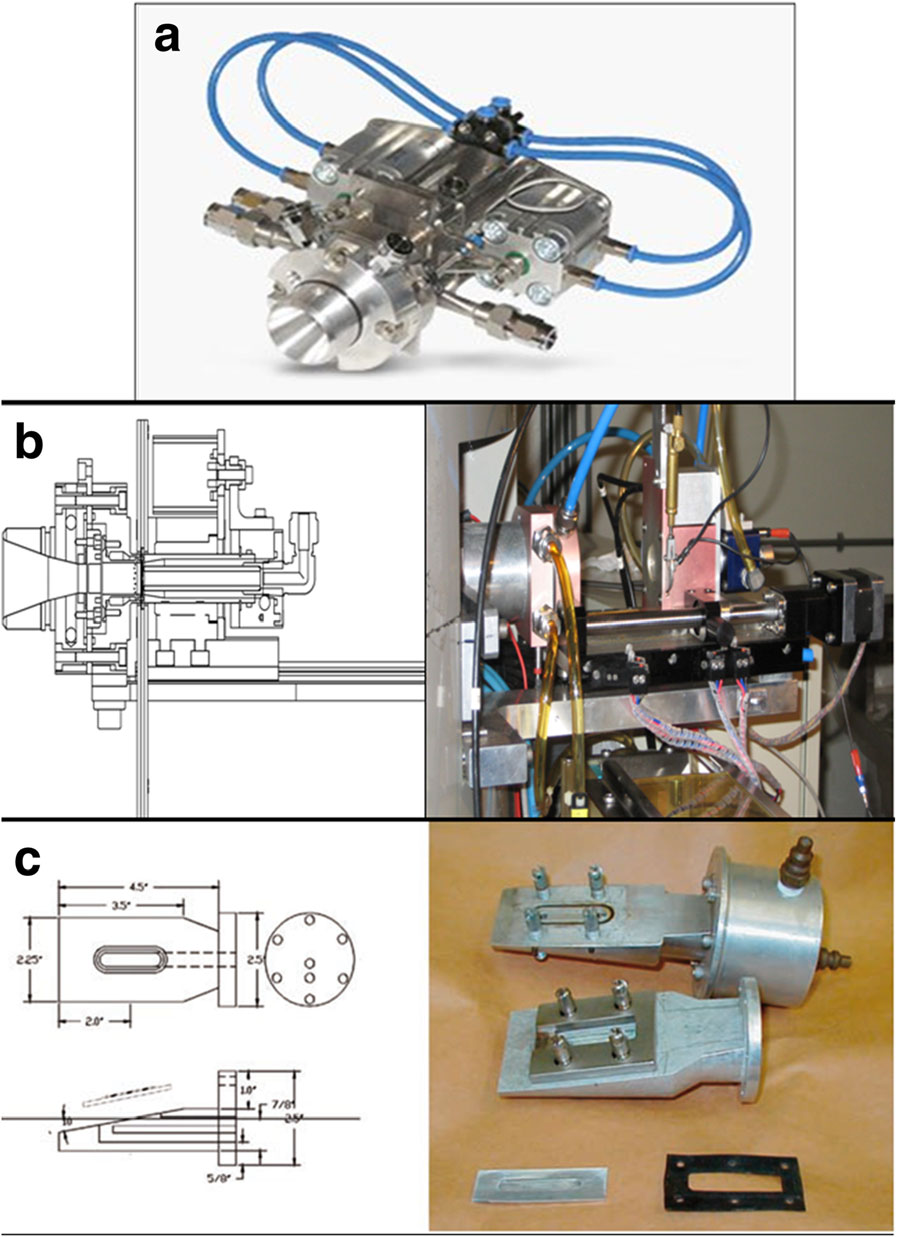
*Examples of solid targets.* **a** COSTIS (Compact Solid Target Irradiation System) IBA Nirta target, **b** Custom developed Zirconium-89 target, images courtesy of Roel Mooij, BV Cyclotron VU, Amsterdam, NL **c** Custom developed inclined solid target, black line depicts beam direction from the cyclotron, cross section visualizes cooling channels (IAEA, Cyclotron Produced Radionuclides: Principles and Practice, 2008)

For novel radionuclides, *solid* targets are often used. Solid targets present a number of difficulties when compared with gas and liquid targets:

1. Often, expensive enriched target materials must be used to produce radionuclides with a high purity;
2. The heat conductivity is much lower, which can lead to overheating problems. Care must be taken to optimize the cooling system and beam parameters to avoid melting of the target. Usually, an inclined target design is proposed with an angle of a few degrees between the incident beam and material layer. This ensures that the entering beam power heat is dissipated over a bigger target area (Fig. 2);
3. The target material layer should be a few hundred μm thick and placed on the back plate made of highly conductive metal. This process requires material deposited isotropically and in a pure form (or sometimes using various oxides). Typically, electrodeposition or powder pressing must be used, followed by sintering. Other methods include powder rolling, laser plating and forming high melting point alloy (Qaim Syed, 2011; Stolarz et al., 2015);
4. Solid state targets require more manual handling which causes a higher radiation hazard for personnel, especially when the target must be retrieved shortly after irradiation. Automation of the delivery line is possible with pneumatic systems;
5. Solid target material in general requires more complex chemical separation steps and further recycling steps.

To overcome overheating problems, low beam currents are used which results in low production yields. Therefore, the beam time is increased to reach useful amounts of radionuclides. Together with chemical and technical challenges, this makes the whole solid target production process time consuming, labor intensive, hazardous in terms of radiation safety and thus expensive.

Many of the solid targets for production of novel radionuclides had to be developed and optimized in-house, which resulted in various and incomparable performances and run parameters when collected between the centers. Design possibilities of those targets often encountered problems related to space limitation caused by cyclotron or target shielding, vault dimensions or technical installations. Nowadays, most cyclotron manufacturers have standardized compact solid target systems available for more popular novel radionuclides. These targets offer easier maintenance, have high automation capabilities and are supplied as a plug-and-play system.

Custom targets have the potential to reach higher yields in comparison to the standard off the shelf compact target (systems). To our opinion this is due to the goal of the custom target developers design their target to be better optimized for their specific conditions of usage and to maximize the yields. On the other hand cyclotron manufacturers with their compact targets, aside for maximizing yields, aim to assure high level of safety, easiness of handling for personnel and introducing more replaceable materials. This situation may create a “trade-off” dilemma for a radionuclide production plant operating a SMC which technology to decide on for a new solid targetry based production.

### Liquid targets for radiometals production

A recent development in the production of radiometals involves the use of *solution targets* (i.e. liquid targets), where the target material is dissolved in an aqueous solution in the form of salts. This method removes the drawbacks that are mentioned for solid targets and offers the following advantages: simple handling, on-demand availability, a faster production process without dissolution and adjustable use of enriched material depending on the needs for a single production, which greatly reduces the cost (Alves et al., 2017). However, the production of radiometals using liquid targets comes with a number of disadvantages, most noticeable the significantly lower production yields. To optimize these methods, certain problems need to be addressed before large-scale implementation.

One of them is the target pressure build-up during irradiation. This effect depends on the beam intensity and concentration of the target material. The irradiation of aqueous solutions leads to water radiolysis and the creation of ions and free radicals of hydrogen, oxygen and hydroxyl groups, which in turn leads to rapid gas evolution in the target chamber (H_2_ and O_2_). This effect is further promoted by the introduction of certain types of salt cations and anions. Those effects were studied and minimized in a recently published patent invented by *DeGrado* et al. (DeGrado, Pandey, and Byrne, 2017). In particular cases, the introduction of strong nitric acid proves to minimize the gas evolution in the target because the nitric acid acts as a free radical scavenger. Another solution is the introduction of a backpressure regulator which keeps the in-target pressure at a stable level during irradiation.

The authors also point out the importance of carefully selecting salt constituents as certain pairs of metallic cations and acidic anions affect the gas evolution in different ways. Additionally, strong acids used in targetry may result in corrosion of the targets. Other experiments using silver or aluminum targets demonstrated the presence of fine particles, leading to clogging of the transfer lines (Hoehr et al., 2012) and thus requiring frequent target maintenance. It is recommended to use heat-resistant and chemically inert materials (such as niobium and tantalum) as target materials. Optimization of target material, metallic salt composition and concentration of acid are all needed to prevent the formation of precipitates. Favorably, the addition of nitric acid also eliminates the precipitation of salts within target (DeGrado et al., 2017).

Pandey et al. show high ratios (approximately 85–90%) of enriched material recycling for liquid targets (Pandey et al., 2014a), which is a highly desirable and financially advantageous factor for considering the use of solution targets.

In general, the liquid target radiometal production technology is not yet mature and requires more studies to optimize production yields. For small facilities and/or facilities at remote locations, the recent liquid target developments allow for in-house production of radionuclides with small, shelf-shielded cyclotrons.

## Developments in radionuclide production

Below, the current status and recent developments in the production of a number of novel radionuclides will be presented.

### Technetium-99m

Technetium-99m (^99m^Tc) is still undeniably the most commonly used radionuclide in the world. Its share in all nuclear medicine procedures is approximately 80%, with an estimated 40 million patient preparations per year worldwide (OECD-NEA, 2017). Until recently, almost all of the ^99m^Tc used in nuclear medicine was produced by radioactive decay of ^99^Mo confined in a generator. ^99m^Tc success in the clinic is based on advantageous physical properties such as: a moderate half-life (T_1/2_ = 6.0 h), and low energy 99% γ emission of single peak of 140.5 keV. Moreover, other beneficial factors for its widespread use are: a low price, availability, and the fact that it is a generator product. Its parent radionuclide, ^99^Mo, is dominantly produced by a nuclear fission process of ^235^U(n,f)^99^Mo in a few aging high neutron flux nuclear reactors running on Highly Enriched Uranium (HEU). The threat of possible shortages in the near future due to imminent closure of those facilities encouraged many research groups to investigate alternative methods for the production of ^99m^Tc without the use of a nuclear reactor (Table 3). Such studies are supported by the International Atomic Energy Agency (IAEA) through the project: “Accelerator based Alternatives to Non-HEU production of Mo-99/Tc-99m” (Accelerator-based Alternatives to Non-HEU production of Mo-99/Tc-99m, 2011).

**Table 3:** ^99m^Tc production pathways

Reaction	Method	Currently available	Status and further development
^235^U(n,f)^99^Mo → ^99m^Tc	Reactor + generator	Worldwide	Well-established, availability will shrink with phasing out of nuclear research reactors
^100^Mo(p,2n)^99m^Tc	Small medical or intermediate energy cyclotron	In Canada	Possible worldwide implementation with decreasing nuclear reactor capacity
^96^Zr(α,n)^99^Mo → ^99m^Tc	α beam cyclotron + generator	No	Will not be implemented. Method not competitive, α-beam required, low yields
^100^Mo(γ,n)^99^Mo → ^99m^Tc	LINAC+ generator	No	Under development in Canada, USA and the Netherlands
^98^Mo(n,γ)^99^Mo → ^99m^Tc	Reactor + generator	In USA and Japan	Auxiliary method used in nuclear reactors (Blaauw et al., 2017; van der Marck, Koning, and Charlton, 2010). Availability will shrink with phasing out of nuclear research reactors
^100^Mo(p,2n)^99m^Tc	Laser	No	Theoretically feasible. Further research required

The concept of producing sufficient amounts of ^99m^Tc from enriched ^100^Mo targets using a cyclotron was known from the early 70’s (Beaver and Hupf, 1971). It was not developed further, since the current capacity of fission produced ^99^Mo for usage in handy ^99^Mo/^99m^Tc generators was sufficient for years to come. In addition, the nuclear reactor network was still expanding. In the 90’s, several researchers again started the investigation of the nuclear data for proton induced reactions for molybdenum radionuclides, most notably ^100^Mo(p,2n)^99m^Tc. The results renewed interest in the production of ^99m^Tc using cyclotrons and set the optimum production energy using protons in the range of 13.5–17 MeV, with a recommendation to avoid higher energies resulting in the production of inseparable impurities such as ^98^Tc, ^97^Tc and ^96^Tc (Manenti et al., 2014; Qaim et al., 2014; Takacs et al., 2016).

Target design became the next milestone for successful cyclotron production of ^99m^Tc, resulting in thick molybdenum coatings for solid targets able to withstand prolonged irradiation of high beam currents accompanied by an easy recovery of ^99m^Tc and recycling of ^100^Mo after end of beam (Stolarz et al., 2015; Hanemaayer et al., 2014). The worldwide leading group for the targetry, beam optimization and ion source studies are undoubtedly Canadian researchers gathered under the auspices of the TRIUMF centre (Schaffer et al., 2015; Hanemaayer et al., 2014; Buckley, 2013; Benard et al., 2014). For the production of technetium-99m, they used solid targets of enriched molybdenum-100 coatings which resulted in a production yield of up to approximately 513 MBq/μAh. This can lead to 350 GBq of ^99m^Tc on an ACSI TR-19 cyclotron (6.9 h beam time, 300 μA beam, cyclotron beam energy lowered to 18 MeV protons) and about 170 GBq on GE PETtrace 880 (6 h beam, modified to 130 μA, 16.5 MeV protons). In the case of 18 MeV proton energy irradiations, which are slightly above the recommended energy range for ^99m^Tc production, the target contained approximately 95% of ^99m^Tc with 99.5% radionuclidic purity at EOB. The separation efficiency of ^99m^Tc from a molybdenum target, which can be easily automated, was proved to be in the range of 80% - 90%. With the radiochemical purity of 99.7% of the final pertechnate [^99m^Tc]TcO_4_ solution, the same USP quality requirements (> 95%) as for ^99^Mo/^99m^Tc generator produced pertechnate are met. The final efficiency of ^100^Mo recycling is above 90%. Other research groups have produced data on similar experiments that concur with these results (Das et al., 2016; Rovais et al., 2016).

Such capacity can fulfill a daily demand for a large area, thus encouraging the Canadian government to co-finance and support The Canadian National Cyclotron Network project, run by ACSI, which will cover 100% of Canadian needs for ^99m^Tc (ACSI, n.d.). The project was scheduled to be finalized before closure of the Chalk River nuclear reactor, once one of the leading suppliers of ^99^Mo, now operating as a back-up plant until its planned closure in March 2018. The project is ongoing, facing several delays caused by regulatory approval issues and logistics considerations (Brown, 2016), but some Canadian nuclear medicine patients are already scanned with cyclotron produced ^99m^Tc.

An interesting alternative pathway of cyclotron production of ^99^Mo has been theoretically examined by a group from the Italian National Institute for Nuclear Physics (Pupillo et al., 2015). They use an α-beam on an enriched Zirconium-96 target, thus inducing a ^96^Zr(α,n)^99^Mo nuclear reaction. The above pathway does not produce sufficient amounts of ^99^Mo for commercial use. However, it has the advantage of an extremely high specific activity in the range of 10^6^ TBq/g (current large generators have a specific activity of up to 370 TBq/g). A limiting factor for the development of this pathway is the unavailability of high current α beams.

Alternative methods can utilize linear accelerators or lasers. The production of molybdenum-99 by utilizing electron induced brehmsstrahlung photons produced in linear accelerators is of particular interest. These targets require the same molydbenum-100 enriched material, but the nuclear reaction pathway is ^100^Mo (γ,n)^99^Mo. Recent experiments in this field confirmed that this production pathway is feasible (Mang'era et al., 2015) and a steady supply of ^99^Mo is expected to be provided soon by the already commissioned 35 MeV, 40 kW Linear Accelerator (LINAC) at the Canadian Light Source. Similar projects based on the same principle are under development or consideration in other countries. NorthStar Medical Radionuclides, USA, is currently finalizing the ^99^Mo production method from the neutron capture ^98^Mo(n,γ)^99^Mo using an old nuclear reactor at the University of Missouri Research Reactor Center (MURR). The company plans to expand its capacity by building a single site LINAC farm, comprised of up to 16 LINACs capable of producing ^99^Mo by a photonuclear reaction on enriched ^100^Mo targets. The project is estimated to cover half of the USA demand for ^99m^Tc (NorthStar Medical Radioisotopes Receives $11.75 Million from National Nuclear Security Administration, 2015; Harvey, Isensee, Moffatt, and Messina, 2014). The Lighthouse Consortium, Netherlands, is gathering companies and research institutes under the leadership of ASML, to achieve their goal of building an electron accelerator facility for ^99^Mo production from enriched ^100^Mo targets. It is assumed that by 2021 the facility will be able to cover the capacity of the HFR nuclear reactor in Petten (Lighthouse: productie medische isotopen vanaf 2021, 2017). In Japan, considerations on which technology to rely on for securing the future ^99m^Tc supply are ongoing. The country already has a greatly developed particle accelerator infrastructure, allowing to diversify between production from linear accelerators, a vast cyclotron network of appropriate energies or even by using the Japan Proton Accelerator Research Complex (J-PARC) spallation facility (Nakai et al., 2014; Fujiwara et al., 2017).

Another alternative is the use of direct laser light that generates proton or electron fluxes further impinging on final targets. The disadvantage of this method is that the particle fluxes are not mono-energetic, which complicates yield calculations for side products. For this reason, a laser facility would require careful optimization of the laser target-nuclear target setup as a whole, to obtain a minimum number of co-produced impurities. Theoretical work in this field has been done with promising results (Bychenkov et al., 2014), yielding 300 GBq of ^99m^Tc with 0.12% radionuclidic impurities in a 6 h irradiation assuming the utilization of the future International Coherent Amplification Network (ICAN) laser concept. However, in comparison to earlier mentioned advancements of particle accelerators, current laser-based ^99m^Tc production research and infrastructure is not developed enough for short-term considerations.

### Radioiodines I-123, I-124

Radioiodines have a long history of usage with changes in favoring different radionuclides throughout the last century (Silberstein, 2012). Today, the widely used ^131^I therapeutic agent (T_1/2_ = 8.02 d, 100% β^−^, 90% β^−^_av_ 192 keV, 82% γ 364 keV) is produced in nuclear reactors. ^131^I is also used for SPECT imaging thanks to its low energy gamma emission.

Iodine-123 is the second most widely used radioiodine and also the second most used imaging agent after ^99m^Tc. Its popularity comes from its availability, perfect γ energy for imaging and appropriate half-life for metabolic studies (Park, 2002). ^123^I (T_1/2_ = 13.2 h, 100% EC, 83% γ 158 keV) and ^124^I (T_1/2_ = 4.18d, 100% EC^+^β^+^, 12% β^+^_av_ 687 keV, 11% β^+^_av_ 975 keV, 63% γ 603 keV) are cyclotron produced and broadly used for all kind of applications (Silberstein, 2012; Koehler et al., 2010). ^123^I is considered a classical radionuclide because of its widespread availability, well-established production method and routine production. Yet it remains an expensive endeavor since the production route utilizes proton irradiations of enriched xenon-124 gas (50,000 $/l as of 2008 (Kakavand et al., 2008)) in closed systems. This method employs two parallel nuclear reactions pathways and the following decay to ^123^I: ^124^Xe(p,2n)^123^Cs → ^123^Xe → ^123^I and ^124^Xe(p,pn)^123^Xe → ^123^I. It also requires an incident proton energy range between 20 and 30 MeV, which applies to a much smaller group of intermediate energy range cyclotrons. Such a produced final ^123^I radionuclide has an excellent radionuclidic purity of 99.9%. It is included in this review due to the availability of an alternative production pathway using enriched Tellurium targets (see later in this section).

Iodine-124 has seen its rise in the first decade of the twenty-first century. Although having a disadvantage of a high energy prompt γ which results in a high patient radiation dose and complicated dose calculations. It emits β^+^, thus this isotope of iodine is fit for PET scans (Silberstein, 2012). Additional Auger electron emission gives it the capability to be named a theranostic agent. Iodine-124 production data is vast since many alternative nuclear production pathways exist, encompassing a big range of reactions based on: ^123-126^Te(p,xn)^124^I, ^123,124^Te(d,xn)^124^I, ^121,123^Sb(α,xn)^124^I and ^123^Sb(^3^He,2n)^124^I nuclear reactions. This irradiation data has been extensively evaluated (Koehler et al., 2010; Braghirolli et al., 2014; Azizakram et al., 2016; Aslam et al., 2010), with the result being an agreement found on the best approach to produce ^124^I by means of an ^124^Te(p,n)^124^I reaction in the range of 14–7 MeV proton energy, with a yield reaching 21 MBq/μAh and minimum ^125^I impurities.

Both ^123^I and ^124^I have the great advantage of proton reaction cross section having a nuclear threshold below 10 MeV on Tellurium targets (Soppera, Bossant, and Cabellos, 2017a). This gives the possibility to produce them with most cyclotrons. The developments in the last 20 years focused on the design of more efficient solid target systems (Kakavand et al., 2008; Mahunka et al., 1996; Al-Yanbawi and Al Jammaz, 2007; Nagatsu et al., 2011; Qaim et al., 2003; Poniger et al., 2012). TeO_2_ based thin layer targets can be used for the production of both iodines using the same target, by changing only the tellurium target isotope to match the reactions ^123^Te(p,n)^123^I or ^124^Te(p,n)^124^. The use of ^123^I and ^124^I remains limited because the raw material cost of enriched tellurium (recyclable) remains high: $10–50/mg (Fonslet and Koziorowski, 2013) which translates directly to the radionuclide price.

### Zirconium-89

Zirconium-89 (T_1/2_ = 78.4 h, 100% EC^+^β^+^, 23% β^+^_av_ 396 keV, 99% γ 909 keV) has been proven very useful in drug development, for instance for new antibody therapies. This is because of its:

1. long half-life that is suitable for studying the biodistribution of long-circulating proteins and antibodies,
2. reproducible applicability in chelating chemistry and conjugation with monoclonal antibodies (mAbs) used in ImmunoPET studies (Vosjan et al., 2010; Ikotun and Lapi, 2011; Rice et al., 2011),
3. balanced physical properties, i.e. sufficiently small β^+^ energy to maintain good image resolution and acceptable patient dose levels.

Zirconium-89 was associated with a low radionuclidic purity of the recovered product from Yttrium-89 target material and chelation chemistry (Holland, Sheh, and Lewis, 2009). The research group of the Vrije Universiteit (VU) University Medical Center in Amsterdam (NL) simplified the method which resulted in reproducible radiolabeling encouraging the worldwide development of the production and use of ^89^Zr (Vosjan et al., 2010).

^89^Zr can be produced in cyclotrons by 3 nuclear reaction pathways:

1. ^nat^Sr(α,xn)^89^Zr
2. ^89^Y(d,2n)^89^Zr
3. ^89^Y(p,n)^89^Zr

The great advantage of producing ^89^Zr from the latter two reactions is the fact that naturally occurring Yttrium is 100% composed of the ^89^Y radionuclide (thus ^nat^Y = ^89^Y). This significantly reduces the cost and availability of the target material in comparison to other radionuclides that require highly enriched materials. The first reaction (^nat^Sr(α,xn)^89^Zr) requires a rarely available α-beam and is also prone to producing impurities from a differentiated strontium isotopic composition (unless enriched) so this pathway is limited to theoretical considerations.

The second reaction (^89^Y(d,2n)^89^Zr), although proven to give high yields both theoretically and experimentally (Sadeghi, Enferadi, and Bakhtiari, 2012; Tang et al., 2016), requires a relatively high energy deuteron beam since its reaction threshold starts at 5.9 MeV and peaks in the range of 13–17 MeV (Soppera, Bossant, and Cabellos, 2017b) which excludes most common small medical cyclotrons. Although the GE PETtrace 800 series is capable of producing a deuteron beam with 8.4 MeV and the IBA Cyclone 18/9 is capable of producing deuterons of 9 MeV, it is too low to yield reasonable amounts of ^89^Zr. Therefore, this pathway is reserved mostly for research centers possessing high energy, multiple beam type cyclotrons.

The third reaction (^89^Y(p,n)^89^Zr) is the only way for a small medical cyclotron facility to provide ^89^Zr. With radiochemical methods developed, recent worldwide research focused on target development to increase the production yields. One of the challenges is the limitation of beam energy, since above the reaction threshold of 13.08 MeV, production of long-lived ^88^Zr(T_1/2_ = 83 d) occurs via the (p,2n) reaction, which is an impurity inseparable from the final product. *Dabkowski* et al. (Dabkowski et al., 2015) used a compact IBA COSTIS target, designed to produce ^89^Zr, to show that above the reaction threshold of 11.6 MeV there is a co-production of another contaminant ^88^Y (T_1/2_ = 107 d) via ^89^Y(p,pn)^88^Y. The yttrium atoms must be separated in the following chemistry process minimizing this amount. Below 10 MeV proton beam energy, the authors reported a yield of insufficient quantities of ^89^Zr. This leaves a narrow beam energy window for practical use with small medical cyclotrons. For 11.6 MeV proton beams, the yields were about 14–16 MBq/μAh with maximum beam currents of 30 μA in 3–3.5 h irradiation time. This gives approx. 1.4 GBq of ^89^Zr at EOB with a high radionuclidic purity.

In the case of custom made targets, it has been reported that one can achieve higher yields than this, while maintaining high radionuclidic purity of the final product (Sadeghi et al., 2012; Tang et al., 2016; Alfuraih et al., 2013). Custom made yttrium target developments published in recent years (Siikanen et al., 2014; Ellison et al., 2016), which encompass welding yttrium foils, modifications in yttrium foil thicknesses and water cooling, report applicable designs capable of delivering yields of up to 49 MBq/μAh with maximum currents of 45 μA, and a 90% separation efficiency. This gives about 1.4–2.2 GBq of radionuclidic pure ^89^Zr in 1–2 h beam time, thus effectively shortening the irradiation times on the cyclotron or increasing the production capacity.

Production of ^89^Zr using solid targets with a small medical cyclotron might be challenging because of workspace limitations. Therefore, *Pandey* et al. (Pandey et al., 2016) and *DeGrado* et al. (DeGrado et al., 2017) have studied a few cases of ^89^Zr production via proton irradiation of liquid targets, filled with a Y(NO_3_)_3_/HNO_3_ solution, and corresponding chemistry. So far, their development has a much lower yield than the solid target technologies, reporting 4.4 MBq/μAh with a 40 μA beam current irradiated for 2 h. This means approx. 370 MBq of ^89^Zr activity with > 99% radionuclidic purity which is sufficient for a very small range of applications (preclinical or a few patients on-site) and in agreement with previous work on liquid target production of ^89^Zr (Oehlke et al., 2015).

### Copper-64

Copper-64 (T_1/2_ = 12.7 h, 42.5% EC, 18% β^+^_av_ 278 keV, 39% β^−^_av_ 190 keV, 0.5% γ 1346 keV) has seen its rise starting in the late 90’s and continuing in the first decade of the twenty-first century. It is one of the commonly used (mostly in the USA) non-standard radionuclides in PET imaging thanks to several advantages (Ikotun and Lapi, 2011). Its half-life allows for shipping to distant centers, its low positron energy and little γ emissions make the image resolution high, and high-yield and high-purity production methods are developed. Moreover, ^64^Cu has an unexplored theranostic potential, since its positron emission is accompanied with β^−^ and abundant auger electron emission. Uses of chelated ^64^Cu complexes are many, such as small molecules, peptides and mABs. However, the latest developments of ^89^Zr possess the potential to take over these applications because of a longer half-life that better matches the biological half-life of the mAbs and it is cheaper to produce.

Production of ^64^Cu is very well established. It is most commonly produced by cyclotrons utilizing a proton beam impinging on an enriched nickel-64 solid target reacting through a ^64^Ni(p,n)^64^Cu channel. The reaction threshold is 2.5 MeV and the highest yields are achieved in the proton energy range of 10–15 MeV, well within the energy range of a small medical cyclotron. There are commercially available solid targets, based on electroplating ^64^Ni on gold discs, which dominate the ^64^Cu production market. In optimal situations, this method can produce up to 185 GBq (Ikotun and Lapi, 2011) after a long beam time of up to 8 h and currents up to 40 μA with various target thicknesses and proton energies applied, but typically lower yields are enough to supply local area customers for a few days (Avila-Rodriguez, Nye, and Nickles, 2007; Matarrese et al., 2010; Szelecsényi, Blessing, and Qaim, 1993). With radionuclide production secured, recent developments in ^64^Cu focus on successful automation of the radiochemistry (Poniger et al., 2012; Matarrese et al., 2010; Ohya et al., 2016) and developments of reliable tracers carrying ^64^Cu (Jalilian and Osso, 2017).

The above method, even though well-established and giving enough high purity material, is not as common as one would expect. It has a number of disadvantages (Alves et al., 2017; Jalilian and Osso, 2017):

- ^64^Ni has a very low abundance (0.95%), which requires significant enrichment before irradiations can be performed, making it an expensive method;
- It requires on-site electroplating equipment and recycling technology;
- Maintaining the suitable quality of electroplating (surface roughness) during long electroplating processes (6 - 48 h) requires careful handling (Rajec et al., 2010);
- Long irradiation times are needed occupying the accelerator;
- The operators can receive a high radiation dose unless an automated process is developed.

Thus, other methods were searched for in order to simplify the whole process. One of the options involved replacing the production pathway with ^67^Zn(p,α)^64^Cu reaction, which was investigated by *Szelecsényi* et al. *(*Szelecsényi et al., 2014*)*. Even though it was proven that this method is feasible for the commercial production of ^64^Cu, it offers radiochemical purity challenges and poses the same concerns regarding high enrichment of the solid target material.

The latest most interesting development, concerns producing ^64^Cu in a liquid target as described by *Alves* et al. (Alves et al., 2017). Such a change would allow for a faster, safer and simplified operation with automated loading and transfer to an accompanied automated chemistry module. The group conducted a series of experiments with enriched ^64^Ni dissolved in the novel IBA liquid target made of niobium. After an irradiation of 5 h, 4.6 GBq of ^64^Cu at EOB was produced, which translates to a yield of 0.14 MBq/(μAh·mg). The authors claim that this can easily be improved by increasing the enriched ^64^Ni concentration and beam time to achieve up to 25 GBq of ^64^Cu. The subsequent automated purification gave radiochemically pure ^64^CuCl_2_ with a 77% decay corrected yield. This amount could satisfy local needs.

### Gallium-68 and Gallium-67

Over the past several years, Positron Emission Tomography (PET) imaging agents labeled with gallium-68 (^68^Ga) have undergone a significant increase in clinical utilization. ^68^Ga is conveniently produced from a germanium-68/gallium-68 (^68^Ge/^68^Ga) generator. Besides gallium-68, two other gallium radionuclides are useful in nuclear medicine applications, namely ^66^Ga and ^67^Ga. Only ^67^Ga, besides ^68^Ga, is used routinely in clinical applications. ^66^Ga (T_1/2_ = 9.5 h, 44% EC^+^β^+^, 51% β^+^_av_ 1904 keV, 37% γ 1039 keV, 23% γ 2751 keV) is rarely used because of its high positron energy which lowers the resolution of images combined with a high patient dose as it emits multiple γ rays of energies above 1 MeV. Currently, sufficient base of evaluated nuclear data exists for the production of these radionuclides (Aslam, Amjed, and Qaim, 2015).

^68^Ga (T_1/2_ = 67.7 min, 100% EC^+^β^+^, 88% β^+^_av_ 836 keV, 3% γ 1077 keV) became the radionuclide of particular interest. Its half-life is relatively short, but it is often irrelevant since the majority of ^68^Ga is available at nuclear medicine departments from ^68^Ge/^68^Ga generators (Germanium has a half-life of 271 days) and the short half-life lowers the patient dose. Together with well-known radiochemistry and kit-based preparation methods, ^68^Ga development accelerated, finding application mostly in peptide-based tracers, antibodies and small research molecules (Jalilian, 2016). It is also a possible candidate for further theranostic use. Many ^68^Ga-labelled tracers are used in clinical trials and some are already approved by local authorities: ^68^Ga-DOTATATE (approved by the US Food and Drug Agency in the USA) and SomaKit TOC™ (approved by the European Medicines Agency) (Brief, 2016; Applications, 2016). For neuroendocrine tumor imaging in the UK, Gallium-68 based PET scans were recently advised as a replacement for ^111^In based SPECT scans (Kalsy and Vinjamuri, 2016).

Radiochemically pure Germanium-68 is produced via several alternative routes, mostly using a ^nat,69^Ga(p,xn)^68^Ge nuclear reaction on higher proton energy cyclotrons of > 20 MeV, capable of delivering beam intensities of up to a few hundred microamperes for up to a few weeks of irradiation time (IAEA, 2010). This requires a robust and complex solid target design that can handle a high heat transfer for prolonged periods, as well as reliable purification of the material once the irradiation is complete. The rising need for ^68^Ga radionuclide fuels developments in this area (Fitzsimmons and Mausner, 2015a; Fitzsimmons and Mausner, 2015b; Sounalet et al., 2014; Bach et al., 2013).

^68^Ge/^68^Ga generators have worldwide coverage. Still, small cyclotron production of ^68^Ga is possible by bombardment by protons on an enriched ^68^Zn or ^nat^Zn target (Engle et al., 2012) since the nuclear reaction of ^68^Zn(p,n)^68^Ga has a high cross section of up to 1 b in the energy range of 11–14 MeV. However, this method cannot compete with the ^68^Ge/^68^Ga generators when it comes to issues such as:

- complex solid target handling and development;
- the need for enriched material;
- acceptance of inseparable impurities of ^66^Ga and ^67^Ga.

The comparison looks different when it comes to direct medical cyclotron productions using liquid targets, allowing for fast, small scale on-site productions of radiochemically pure ^68^Ga. Such a method, based on a ^68^Zn(p,n)^68^Ga reaction, would be of particular benefit for hospital cyclotron facilities with spare capacity to add a ^68^Ga-tracer to their routine operations. To achieve a high purity of ^68^Ga from direct irradiation of enriched ^68^Zn, the proton beam energy must be optimized since the threshold for producing the ^67^Ga contaminant from competing ^68^Zn(p,2n)^67^Ga is 12 MeV, so in the peak area of the ^68^Ga production cross-section. Works toward achieving liquid target technology were initiated by *Pandey* et al., the same group that was mentioned before for the development of Zr-89 liquid target (Pandey et al., 2014b). They proposed a dissolution of enriched Zn^68^ target material in nitric acid. The authors claim that they achieved a cheap method for producing small quantities of radiochemically pure ^68^Ga for 2–4 patients in a beam time of approx. 1 h. Furthermore, a recent study using a novel IBA target was carried out by *Alves* et al. (Alves et al., 2017), the same research publication that described the possibilities of liquid target ^64^Cu production. The results look very promising. A 45 min irradiation of 30 mg/ml of ^68^Zn with a 45 μA proton beam yielded 6 GBq of ^68^Ga, which can be translated to achieving batches of 40 GBq of ^68^Ga (pre-purification) by optimization of concentration and beam parameters. This can create a viable alternative for solid target and generator produced ^68^Ga. Besides general advantages of using a solution target instead of solid target, no long lived ^68^Ge impurities were found in the final product. Authors of both above mentioned works also motivate their studies with the need to prevent ^68^Ge breakthrough that occurs in generators, but this argument seems less relevant since today’s modern generators have included mechanisms that nullify that effect (Roesch, 2012a).

Gallium-67 (T_1/2_ = 78.3 h; 100% EC; 39% γ 93 keV, 21% γ 185 keV, 17% γ 300 keV) is a less widely used SPECT imaging agent, but has a potential for broader usage. Its most notably used tracer worldwide is ^67^Ga citrate in various inflammatory studies leading to the detection of tumors and infections (Cwikla et al., 1999; Jalilian et al., 2009). ^67^Ga could also become a therapeutic agent in the future due to the emission of Auger electrons.

Several possible pathways for the production of ^67^Ga exist from proton to deuteron (Tárkányi et al., 2004) and alpha beams. The most commonly used method is proton irradiation of solid targets of either enriched or natural zinc, inducing a ^68^Zn (p,2n)^67^Ga reaction (reaction threshold 10.3 MeV) and a ^67^Zn(p,n)^67^Ga reaction (reaction threshold 1.8 MeV) (Aslam et al., 2015; Asad et al., 2014). ^68^Zn(p,2n)^67^Ga requires a higher energy cyclotron as the reaction cross-section peaks around 21 MeV. It will inevitably lead to the co-production of large amounts of ^68^Ga, creating the need to wait until this impurity level is reduced to acceptable limits. 50 GBq was reportedly produced in one batch with this method (Qaim, 2012). Since ^67^Ga is the longest lived of gallium radionuclides and waiting time for removal of impurities is applied, this method can also use natural zinc targets, considerably reducing costs and employing both reaction pathways. Such a method was presented by *Martins* et al. (Martins and Osso, 2013) where the authors investigated a new ^67^Ga purification technique, resulting in radionuclidic pure (99.9%) ^67^Ga production yields of 40 MBq/μAh with a 26 MeV proton beam on a natural zinc solid target. One must also consider unavoidable losses connected to the extraction efficiency (72%) and decay period of 3 days.

Channel ^67^Zn(p,n)^67^Ga offers an alternative for use in small medical cyclotrons that do have enough proton beam energy. To make it efficient, one must consider expensive enrichment since the natural abundance of ^67^Zn is very low (4%). Staying below the 13 MeV production threshold for ^66^Ga will not require unnecessary decay-out time for the by-products. Even though this method is theoretically feasible, it is not commercially implemented. To our opinion, this is due to the fact that the long half-life of 3.3 days allows for an easy distribution of this nuclide. A single intermediate energy cyclotron may easily cover regional needs, so implementing a time-consuming, solid, enriched target ^67^Ga production process on a small medical cyclotron has little economical justification. Perhaps with further increasing demand for this nuclide, small scale production sites will be needed.

### Indium-111

Indium-111 (T_1/2_ = 67.31 h; 100% EC; 94% γ 245 keV, 91% γ 171 keV) is one of the classical radionuclides used in SPECT studies, thanks to its favorable low-energy and high-intensity emissions, and half-life suitable for in vivo studies. Possible medical uses are enormous (Lahiri, Maiti, and Ghosh, 2013), with monoclonal antibodies labeling, blood cell labeling for migration studies, tumor imaging, diabetes studies and more. ^111^In can also be considered an Auger electron therapy agent (Qaim, 2012). ^111^I is not only used in nuclear medicine, but also in material science(Lahiri et al., 2013).

Currently, the most common production route for ^111^In is via proton irradiation of highly enriched Cadmium-112 solid targets, through reaction pathway ^112^Cd(p,2n)^111^In. It is carried out by intermediate energy cyclotrons with protons of an energy range of 25 MeV, with the peak reaction rate around 20–22 MeV (cross section approx. 1000 mb). This method offers high yields (248 MBq/μAh) with co-produced impurities of ^112^Cd and ^112^Sn, which are later separated. Batches of 50 GBq are reportedly produced on a regular basis (Qaim, 2017).

Despite being a well-established classical cyclotron produced radionuclide, it can be produced using novel methods employing SMC. From this point of view, only nuclear reaction ^111^Cd(p,n)^111^In is feasible (Alipoor, Gholamzadeh, and Sadeghi, 2011). It peaks at 15 MeV with approximately 800 mb reaction cross section. A theoretical yield of 67.5 MBq/μAh is expected, which is 4 times less than the one used in the intermediate energy range cyclotrons. An advantage is that up to 20 MeV no considerable co-production of isotopic impurities is expected. However, no literature on such experimental production attempts were found. It is doubtful that this method would find enough interest among small medical cyclotron users, as the radionuclide is readily available in sufficient amounts.

### Yttrium-86

Yttrium-86 (T_1/2_ = 14.7 h, 100% EC^+^β^+^; 12% β^+^_av_ 535 keV, 6% β^+^_av_ 681 keV, 83% γ 1077 keV) can be considered a very special PET radionuclide. If it was not for the widely used therapeutic agent ^90^Y (T_1/2_ = 2.67d, 100% β^−^_av_ 934 keV), ^86^Y would probably not receive any special attention. Its positrons have relatively low intensity and high energy for imaging in proper resolution. However, there is a big advantage of being the surrogate of the therapeutic nuclide – it can be used as a biodistribution imaging agent with the use of the same tracers, with already implemented labeling methods. This is especially important as ^90^Y is a pure β^−^ emitter, offering only very limited SPECT imaging due to brehmsstrahlung effect. A ^86^Y/^90^Y labeled mixture administered to the patient is a good example of a theranostic application, allowing simultaneous therapy with PET imaging (Rösch, Herzog, and Qaim, 2017).

*Zaneb* et al. (Zaneb et al., 2015) has theoretically proven that ^86^Y is best produced on small medical cyclotrons using a ^86^Sr(p,n)^86^Y nuclear reaction. It is predicted that the yield for this reaction is for 371 MBq/μAh assuming 100% enrichment of the solid target and irradiation by 14 MeV protons. The main competitive method against it is ^88^Sr(p,3n)^86^Y, which offers an almost 3-fold higher integral yield (1005 MBq/μAh), but requires an energy range of 43–33 MeV. The advantage of the latter process is that ^88^Sr is a much more abundant stable isotope of strontium (82.5% compared to 9.7% for ^86^Sr), which can translate into a lower cost of enriched materials. However, the high energy range still requires a rare high energy cyclotron, and there are serious considerations with impurity levels of ^87m^Y, ^87m^Y, and ^85^Y. The two factors combined render the second method uninteresting.

Production via the ^86^Sr(p,n)^86^Y reaction can give a product of high radionuclidic purity. A highly present by-product is ^86m^Y (T_1/2_ = 48 min), but it decays to ^86^Y which turns out to simply increase nuclide production after EOB and should not be considered an impurity. Target post irradiation cooling and post processing of the nuclide considerably lower the amount of ^86m^Y at the End of Synthesis (EOS). *Reischl* et al. (Reischl, Roesch, and Machulla, 2002) irradiated a 95.6% enriched solid ^86^SrCO_3_ target with 15.1 MeV protons which resulted in average production yields of 48 MBq/μAh with a radionuclidic purity of > 99% at EOB. The later separation by electrolysis (1 h) gives separation yields of 97%, no carrier-added. Using a 10 μA beam over 2.5 h, this results in 1 GBq of purified ^86^Y. *Yoo* et al. (Yoo et al., 2005) achieved the same separation yield and radionuclidic purity using a very similar setup. They had the same enrichment of ^86^Sr and used electrolysis for separation (with different parameters taking up to 3 h). A different target was used, namely SrO, and 2 μA, 14.5 MeV proton beams for 2 h resulted in much higher yields of 166 MBq/μAh. A few years later, a study by *Avila-Rodriguez* et al. (Avila-Rodriguez, Nye, and Nickles, 2008) revealed a faster separation method using filtration that takes only 20 min. They used several 11 MeV proton beams of 10 μA for 2 h using a 97% enriched strontium ^86^SrCO_3_ target. Even though this method is faster, it has a lower separation efficiency of 88%, results in carrier presence in the order of tens of ppm’s and has a lower radionuclidic purity of 97% at EOB (excluding ^86m^Y) with 2.5% ^87m^Y. The yield was 44 MBq/h – so in the same range as *Reischl* et al. (Reischl et al., 2002) – which confirms the superiority of SrO targets used by *Yoo* et al. (Yoo et al., 2005). ^86^Sr was fully recyclable in all cases.

The above mentioned works result in delivering an approximate amount of 1 GBq of ^86^Y labeled tracer which, in conjunction with ^90^Y therapy agents, could satisfy the need for a few patients.

An experiment is reported on ^86^Y production using liquid targets. *Oehlke* et al. (Oehlke et al., 2015) irradiated a 0.9 ml Niobium-body Havar-window liquid target with a 13 MeV proton beam from a small medical cyclotron. The solution contained ^nat^Sr (NO_3_)_2_ salt dissolved in ultrapure water. Beam conditions were 4.6 μA in average, running for 1 h. The separation using DGA resin was 99% efficient. The resulting yield was 1.44 MBq/μAh, considerably lower than the above mentioned solid target results, but it is worth mentioning that natural strontium was used with a 10 times lower content of ^86^Sr than enriched material. By doing a simplified estimation, using an enriched material could give us an increase in yield of 10 times to 14 MBq/μAh. Small medical cyclotrons can have 2–3 higher target volumes than reported here – a further increase of the target volume could provide another yield increase. There is also room left to adjust the concentration of the salt. In conclusion, a liquid target method is a possible alternative for easier production of Yttrium-86 on a small scale.

### Scandium-44

One of the very recent radionuclides of interest for PET imaging is ^44^Sc (T_1/2_ = 3.97 h, 100% EC^+^β^+^, 94% β^+^_av_ 632 keV,100% γ 1157 keV). Due to the chemical similarity of Sc^3+^ cation to Lu^3+^ and Y^3+^, DOTA complexes labeled with those radionuclides show very similar properties in vivo. This makes it a possible diagnostic surrogate for therapeutic tracers containing ^177^Lu or ^90^Y. Currently, [^68^Ga]Ga-DOTA-complexes are used for this purpose (Krajewski et al., 2013). ^44^Sc shows superiority over Gallium-68, given that the routine production method is well established. It does not require an expensive ^68^Ge/^68^Ge generator, it has a half-life suitable for logistics, a lower positron energy and slightly higher β^+^ branching ratio. Moreover, ^47^Sc (T_1/2_ = 3.35 d, 100% β^−^, 69% β^−^_av_ 143 keV, 31% β^−^_av_ 204 keV, 68% γ 159 keV) is a therapeutic radionuclide of rising interest, which creates possibilities for a theranostic agent by applying a matched pair of ^44^Sc/^47^Sc. ^44^Sc can also be used independently in peptide based imaging, as well as antibody labeling and small protein labeling (Hernandez et al., 2014).

Multiple methods for the production of ^44^Sc have been investigated. Experiments were done with an α- beam at 29 MeV (Szkliniarz et al., 2016), a deuteron beam at 16 MeV (Alliot et al., 2015) and an innovative method employing a ^44m^Sc/^44^Sc in vivo generator (Huclier-Markai et al., 2014). In the latter case, the in vivo generator utilizes the existence of the metastable state of ^44m^Sc (otherwise treated as impurity) that decays by internal transition (98.8%) to ^44^Sc, which later emits positrons used for imaging. This way, the long half-life of ^44m^Sc (T_1/2_ = 58.6 h) allows for longer pharmacokinetic studies, which is especially important when dealing with mABs. Additionally, ^44m^Sc is closer with its half-life to the therapeutic isotope ^44^Sc (2.44 vs 3.35 d), making it possible to monitor drug metabolism over a longer period of time. *Huclier-Markai* et al. (Huclier-Markai et al., 2014) prove that the recoil energy of the decay of the metastable state, which is only 271 keV above its daughter state of ^44^Sc, is too low (0.89 eV) for the isotope to leave the chelator molecule, thus assuring stability of the complexes used.

Another method to acquire ^44^Sc is the ^44^Ti/^44^Sc generator (Roesch, 2012b; Filosofov, Loktionova, and Rösch, 2010). There, the parent nuclide of ^44^Ti (60.6 y, 100% EC, 93% γ 68 keV, 96% γ 78 keV) decays by electron capture to ^44^Sc for possible elution. The concept is based indirectly on the usage of cyclotrons, as the ^44^Ti is produced by a ^45^Sc(p,2n)^44^Ti reaction. An especially advantageous factor of this method is that ^45^Sc is the only naturally occurring stable isotope of scandium; therefore, there is no need to acquire expensive enriched material for irradiations. The peak cross section for this reaction is around 20 MeV proton energy (Soppera et al., 2017a), but the ^44^Ti production method is inappropriate for utilization by SMCs as production of very long-lived ^44^Ti would require a relatively high proton beam current, which is typically not possible with SMCs. For this reason, *Filosofov* et al. [104]produced 185 MBq of ^44^Ti on an intermediate energy cyclotron with proton energies of 25 MeV and a beam current of 200 μA. The final proposed generator allowed them to elute 180 MBq of Scandium on a weekly basis, which was continued for a year. The authors report a negligible amount of ^44^Ti breakthrough in the order of 10^− 5^ Bq. However, additional post processing steps are required to obtain a ^44^Sc solution. Additionally, long elution intervals and little amounts of final product are a certain limitation of the method, which can be countered only by intensive irradiations that could increase the amount of ^44^Ti.

In the case of small medical cyclotrons the common production route is via the ^44^Ca(p,n)^44^Sc nuclear reaction by using a solid target irradiation of highly enriched ^44^CaCO_3_ powder. The reaction cross section maximum reaches approx. Eight hundred mb for 11 MeV protons. ^44m^Sc (T_1/2_ = 58.6 h, 99% IT, 87% γ 271 keV) is an impurity of concern, although the energy of the γ does not carry a risk to patients or the PET image resolution. Its long half-life creates the effect of the in-vivo generator mentioned before. The reaction ^44^Ca(p,n)^44m^Sc cross-section reaches almost 100 mb and peaks at 13 MeV.

*Krajewski* et al. (Krajewski et al., 2013) presented an optimization study on the ^44^Ca(p,n)^44^Sc reaction pathway which reports 9 MeV to be the best optimized proton energy, giving > 99% radionuclidic purity with only 0.09% ^44m^Sc content in the finished product. Other impurities of ^43^Sc, ^46^Sc, ^47^Sc and ^48^Sc were negligible or not detectable at all. 4 h irradiation of 20 mg of enriched [^44^Ca]CaCO_3_ with 12,4 μA beam resulted in 2.5 GBq of ^44^Sc. The further separation yield was 70%, calcium content measured by ICP-MS analysis was below 1 ppm. Further labeling yield with DOTA-TATE was > 98%, and the target recycling efficiency was only 60%. Other research groups have reached higher separation efficiencies of 80% (Valdovinos et al., 2015), and high radiochemical yields with different tracers above 90% (Hernandez et al., 2014), but their use of natural calcium as a target limits the production yield and radionuclidic purity, which in turn is not of use in clinical applications.

Recently published work by *Meulen* et al. (Meulen et al., 2015) presented a more efficient process and confirmed that the low energy range gave the best results, with an 11 MeV proton beam on 10 mg of 97% enriched ^44^CaCO_3_ target. With an improved target design, they were able to irradiate with 50 μA, hence using a shorter time of 90 min, reaching 1.9 GBq of ^44^Sc at EOB (note the amount of irradiated material is halved in comparison to the above study (Krajewski et al., 2013)). Moreover, the group reports a faster and very efficient separation method with 98% efficiency with < 1 ppm metallic contaminant levels. Radionuclidic purity was 99% and further radiolabelling efficiencies with DOTANOC were > 98%. The study also showed great improvement in the recycling of enriched calcium reaching 98% of initial enriched material recovery, significantly reducing the operational costs in comparison to work of *Krajewski* et al. (Krajewski et al., 2013). The required proton energy is low enough for almost all small medical cyclotrons, provided that the use of the solid target is accepted. The disadvantage of the method is certainly the cost of ^44^Ca. Its natural abundance is only 2.1%, therefore 97% enriched ^44^CaCO_3_ costs about $15/mg (Meulen et al., 2015). Fortunately, the material is highly recyclable.

^44^Sc was also investigated by *Oehlke* et al. for the production in a liquid target (Hoehr et al., 2014). Thirteen MeV proton beam irradiated natural calcium nitrate Ca (NO_3_)_2 in_ dissolved in ultrapure water, in a relatively small volume target of 0.9 ml. The authors report a maximum of 28 MBq at EOB with a 20 μA beam current for 1 h, which may seem disappointing from a clinical perspective. However, a number of alterations could prove the method to be viable such as: increasing the beam time, target volume and concentration, and most importantly, a high enrichment of ^44^Ca could elevate the produced activity to GBq level.

### Less common radionuclides in small medical cyclotron energy range

There are many other non-standard radionuclides that can be produced using small medical cyclotrons, but currently the usage of them is limited, either because of a lack of developed applications, scarce nuclear data, or significant investment needs. In a recent, impressive work by *Qaim* (Qaim, 2017), a list of such nuclides is presented. Less common diagnostic purpose radionuclides of increasing interest are especially: ^51^Cr, ^45^Ti, ^76^Br, ^90^Nb or ^94m^Tc.

Therapeutic radionuclides are mostly produced in nuclear reactors and, due to their nature, are most often β^−^ emitters. These are, in general, neutron rich elements. However, there are also pathways possible for the production of therapeutic radionuclides using small medical cyclotrons such as ^67^Cu, ^186^Re, ^103^Pd and ^225^Ac (Qaim, 2017), but those nuclides are reported to be very expensive (Zimmermann, 2013).

## Conclusions

This review presented the developments in small medical cyclotron production of radionuclides used for diagnostics over the past few years (Table 4). Some are increasingly being used in nuclear medicine, i.e. ^68^Ga and ^89^Zr. Some have a fast clinical introduction, taking over the field already occupied by previously implemented methods such as: cyclotron produced ^99m^Tc over previous molybdenum generator supplied ^99m^Tc; ^89^Zr over ^64^Cu in antibody labeling mostly due to a better suited half-life and no enrichment. And lastly, some novel radionuclides, like ^86^Y or ^44^Sc, present promising data for production implementation using SMCs, while gaining interest in clinical applications.

**Table 4:** Comparison of the properties of presented radionuclides

Radionuclide (in order of appearance)	Imaging procedure	T_1/2_	I	E_γ_ or E_av. β_ (keV)	Feasible SMC (< 20 MeV) nuclear reaction	Target type	Yield
^99m^Tc	SPECT	6.43 h	γ 99%	140.5	^100^Mo(p,2n)^99m^Tc	Solid	513 MBq/μAh(Benard et al., 2014)
^123^I	SPECT	13.2 h	γ 83%	158	^123^Te(p,n)^123^I	Solid	No data
^124^I	PET	4.18 d	β^+^ 12% β^+^ 11% γ 63%	687 975 603	^124^Te(p,n)^124^I	Solid	21 MBq/μAh (Braghirolli et al., 2014)
^89^Zr	PET	78.4 h	β^+^ 23% γ 99%	396 909	^89^Y(p,n)^89^Zr	Solid Liquid	49 MBq/μAh (Siikanen et al., 2014) 4.4 MBq/μAh (Pandey et al., 2016)
^64^Cu	PET	12.7 h	β^+^ 18% β^−^ 39% γ 0.5%	278 190 134	^64^Ni(p,n)^64^Cu	Solid Liquid	304 MBq/μAh (Qaim, 2017) 0.14 MBq/(μAh·mg) (Alves et al., 2017)
^68^Ga	PET	67.7 min	β^+^ 88% γ 3%	836 1077	^68^Zn(p,n)^68^Ga	Solid Liquid	No data 1.5 MBq/(μAh·mg) (Alves et al., 2017)
^67^Ga	SPECT	78.3 h	γ 39% γ 21% γ 17%	93 185 300	^68^Zn(p,2n)^67^Ga ^67^Zn(p,n)^67^Ga	Solid	No data
^111^In	SPECT	67.3 h	γ 94% γ 91%	245 171	^111^Cd(p,n)^111^In	Solid	67.5 MBq/μAh (Alipoor et al., 2011)
^86^Y	PET	14.7 h	β^+^ 12% β^+^ 6% γ 83%	535 681 1077	^86^Sr(p,n)^86^Y	Solid Liquid	166 MBq/μAh (Yoo et al., 2005) 1.44 MBq/μAh (Oehlke et al., 2015)
^44^Sc	PET	3.97 h	β^+^ 94% γ 100%	632 1157	^44^Ca(p,n)^44^Sc	Solid Liquid	25 MBq/μAh (Meulen et al., 2015) 1.4 MBq/μAh (Hoehr et al., 2014)

Certainly, there are still many concerns for commercializing these novel cyclotron produced radionuclides. Apart from regulatory, financial, pharmaceutical and chemical concerns, there are also technical concerns. These include low production yields, costs of enriched materials, and improvement of solid target methodology, which is time consuming. In our opinion, more work should be published that encompasses production and optimization of non-standard radionuclides using standardized types of compact solid target systems per specific radionuclide. We would like to encourage the commercial compact solid target system users to share their production results and parameters. An alternative method employs the use of liquid targets for this purpose. A number of studies were reported in this paper on the production of novel radionuclides using liquid targets, but so far, the yields reached were mostly too low for commercial implementation. Addressing this issue still requires extensive research in the field of liquid targets used for the production of novel radionuclides.

## Abbreviations

ACSI: Advanced Cyclotron Systems Incorporated
COSTIS: Compact Solid Target Irradiation System
EOB: End of bombardment
GE Healtcare: General Electric Healthcare
GMP: Good manufacturing practice
HEU: Highly enriched uranium
IAEA: International Atomic Energy Agency
IBA: Ion Beam Applications
ICAN: International Coherent Amplification Network
J-PARC: Japan Proton Accelerator Research Complex
LINAC: Linear accelerator
mAbs: Monocolonal antibodies
MURR: University of Missouri Research Reactor Center
PET: Positron emission tomography
SMC: Small medical cyclotron
SPECT: Single photon emission computed tomography
VU: Vrije Universiteit Amsterdam
